# Resveratrol Ameliorates Diabetic Peripheral Neuropathy via the AMPK/mTOR/Bcl‐2 Axis: Integrative Evidence From Network Pharmacology, Mendelian Randomization, and Binding‐Site Evolutionary Constraint

**DOI:** 10.1155/humu/4524272

**Published:** 2026-06-11

**Authors:** Cheng Zhang, Guokang Mo, Yongxing Zhang, Hao Xu, Xiangjun Hu, Jian Zhang

**Affiliations:** ^1^ Department of Orthopedics, Zhongshan Hospital Fudan University, Shanghai, China, fudan.edu.cn; ^2^ Department of Rehabilitation Medicine, Zhongshan Hospital Fudan University, Shanghai, China, fudan.edu.cn

## Abstract

**Background:**

Diabetic peripheral neuropathy (DPN) is a common diabetic complication with unclear pathogenesis. Current therapies are limited, and resveratrol shows potential in DPN but its therapeutic mechanisms remain unclear.

**Methods:**

Candidate targets and pathways were identified via network pharmacology and protein–protein interaction analysis. Two‐sample MR was performed using eQTL and GWAS data to assess causal associations of PRKAA1, PRKAA2, MTOR, and BCL2 with DPN. Molecular docking was used to characterize ligand‐target interactions, followed by mutational constraint analysis based on gnomAD to evaluate evolutionary conservation of binding‐site residues. Functional validation was conducted in streptozotocin (STZ)‐induced DPN mice and high‐glucose‐treated RSC96 Schwann cells and RAW264.7 macrophages, assessing nerve function, oxidative stress, apoptosis, and macrophage polarization.

**Results:**

Resveratrol significantly improved motor nerve conduction velocity, sensory function, and sciatic nerve integrity in DPN mice, while reducing oxidative stress and neuronal apoptosis. It promoted M2 macrophage polarization, with increased IL‐10 and decreased TNF‐*α* levels. Network analysis identified the AMPK/mTOR/Bcl‐2 axis as a central pathway. MR supported MTOR as a risk factor and BCL2 and PRKAA1 as protective factors for DPN. Mutational constraint analysis revealed marked depletion of missense variants and higher pathogenicity scores in resveratrol‐binding residues of MTOR and BCL2, indicating strong evolutionary constraint. Integration of genetic, structural, and functional evidence consistently prioritized MTOR and BCL2 as key targets.

**Conclusions:**

Resveratrol alleviates DPN by modulating oxidative stress, apoptosis, and neuroinflammation via the AMPK/mTOR/Bcl‐2 axis and macrophage polarization. The integration of causal inference and binding‐site evolutionary constraint strengthens target validity and highlights resveratrol as a multitarget therapeutic candidate.

## 1. Introduction

Diabetic peripheral neuropathy (DPN) is one of the most common and disabling chronic complications of diabetes, which can be described as distal symmetric sensory loss, neuropathic pain, and progressive axonal degeneration that significantly impair the quality of life and predisposes gangrene to ulceration and amputation [1, 2]. No disease‐modifying therapy for DPN has been approved despite improvements in glycemic control. Clinical management is based on a combination of risk‐factor management and symptomatic analgesics, imparting only partial improvements [2, 3] and creating a need to remain consistent with multitarget interventions capable of targeting the multifaceted pathophysiology of DPN [4].

Genetic variations in MTOR and BCL2 are closely associated with the susceptibility and progression of DPN, and may affect protein function, signaling activity, and therapeutic response. The pathogenesis of DPN involves convergent metabolic and inflammatory insults, including chronic hyperglycemia‐driven mitochondrial dysfunction, redox imbalance, activation of pro‐inflammatory signaling pathways, microvascular insufficiency, and Schwann‐cell/myelin injury that together culminate in axonal damage [5, 6]. Schwann cells and macrophages both contribute critically to the progression of DPN. Two interacting processes seem especially critical, including impaired energy metabolism in Schwann cells that increases oxidative stress and impaired axonal support, and maladaptive macrophage activation in the peripheral nerve, characterized by a switch toward the pro‐inflammatory M1 phenotype that maintains neuroinflammation by continuing the production of cytokines [7, 8]. Conversely, resolution of inflammation and tissue repair are linked to the activation of M2 macrophages which are alternatively activated. Furthermore, adequate glycemic control contributes to alleviating nerve injury and improving therapeutic efficacy. Notably, oxidative stress, apoptotic signaling, and neuroinflammation can form a feed‐forward loop that accelerates peripheral nerve injury, suggesting that therapeutic strategies simultaneously restoring Schwann‐cell metabolic homeostasis and rebalancing macrophage polarization may provide effective disease modification in DPN.

To verify the druggability and evolutionary conservation of key target binding sites, we performed binding‐site evolutionary constraint analysis, which helps identify stable and reliable targets for resveratrol (Res) against DPN.

Res is a structurally defined stilbene‐type polyphenol with broad antioxidant, anti‐inflammatory, and metabolic regulatory activities. It is abundant in *Reynoutria japonica* (syn. *Polygonum cuspidatum*, Hu‐Zhang) and other botanical sources. Mechanistically, Res activates the SIRT1/AMPK axis, improves mitochondrial function, and modulates apoptotic pathways in multiple disease models [9–11]. In experimental models of diabetes, Res has been reported to attenuate oxidative stress and inflammatory signaling and to improve neural function, suggesting disease‐relevant pleiotropy for DPN [12, 13].

Given the multifactorial nature of DPN, network pharmacology offers a systems‐level, hypothesis‐generating approach to prioritize candidate targets and pathways for multitarget agents and to guide subsequent experimental validation. In the present study, we combined network pharmacology with in vivo and in vitro experiments to study the protective effects and mechanisms of Res against DPN. We hypothesized that Res would ameliorate DPN by reducing reactive oxygen species (ROS) and apoptosis, and by reprogramming macrophage polarization toward an anti‐inflammatory phenotype through activation of the AMPK/mTOR/Bcl‐2 signaling axis. Together, our findings highlight Res as a mechanistically grounded multitarget candidate for intervention in DPN.

## 2. Methods

### 2.1. Animals and Ethics

Eight‐week‐old C57BL/6 mice were used for the research. Animals were kept under specific pathogen‐free (SPF) conditions at Youshu Life Technology (Shanghai) Co. Ltd., with five mice in every cage. Experimental procedures were approved by the Institutional Animal Care and Use Committee of Youshu Life Technology (Shanghai) Co. Ltd. (approval no. Ys‐m202510003). All experimental mice were male C57BL/6 mice, to avoid the interference of estrogen cycle fluctuation and hormonal differences on nerve pain sensitivity and glucose metabolism. All reagents and kits utilized are included in Table S1.

### 2.2. Induction of Type 1 Diabetes and DPN

Type 1 diabetes was induced by intraperitoneal injection of streptozotocin (50 mg/kg) dissolved in 0.1 M citrate buffer (pH 4.5) one time daily for 5 days consecutively. Fasting blood glucose (FBG) was determined 14 days after the last injection; mice with FBG ≥ 16.7 mmol/L were considered diabetic. DPN was assessed at 8 weeks postinduction using behavioral tests as previously described [14].

### 2.3. Experimental Groups and Drug Administration

Mice were randomly assigned to five groups (*n* = eight for all the groups): control (Con), DPN, DPN + Res‐L (50 mg/kg), DPN + Res‐H (100 mg/kg), and DPN + ALA (60 mg/kg). Res and (R)‐(+)‐*α*‐lipoic acid were dissolved in 0.5% carboxymethyl cellulose sodium and administered by oral gavage once daily for 8 weeks (Weeks 8–16). Vehicle (0.5% CMC‐Na) was administered to Con and DPN groups. The dose amount was ≤ 10 mL/kg. Body weight and FBG were monitored monthly. Behavioral tests were performed at Weeks 0, 4, 8, 12, and 16, and motor nerve conduction velocity (MNCV) was measured at the study endpoint. Sciatic nerves were harvested for histological analysis and biochemical assays. Behavioral assessments of mechanical allodynia and thermal hyperalgesia were selected as core endpoints, since sensory hypersensitivity and pain are the most prominent clinical manifestations of early DPN and are classic indicators for evaluating peripheral nerve sensory function.

### 2.4. Behavioral and Functional Assessments

Mechanical allodynia was assessed using von Frey filaments and the up–down method to determine the 50 percent paw withdraw [15]. Thermal nociception was measured by a Hargreaves apparatus with a pre‐established cut‐off time in order to avoid tissue damage [16]. The electrophysiology recording system was used to measure sciatic MNCV in anesthetized mice; body temperature should be maintained at 36°C–37°C during this process.

### 2.5. Histological Evaluation

The samples of the sciatic nerves underwent routine histology treatment, were intergrated in paraffin, and cross‐sectioned to be stained with H&E, Masson′s trichrome and Luxol Fast Blue to measure general morphology, collagen deposition, and myelin integrity, respectively. Images were acquired through the bright‐field microscopy under standard conditions.

### 2.6. Enzyme‐Linked Immunosorbent Assay (ELISA)

Homogenates of the sciatic nerve were made in ice‐cold PBS containing protease inhibitor.Levels of TNF‐*α* and IL‐10 were determined through commercial mouse ELISA kits based on the manufacturer′s instructions. Absorbance was measured at 450 nm, and concentrations were normalized to total protein content determined by BCA assay.

### 2.7. Cell Culture and Treatments

RAW264.7 and RSC96 cell lines were cultured in high‐glucose DMEM supplemented by 10% FBS and 1% penicillin‐streptomycin at 37 C under 5% CO_2_. The cytotoxic concentrations of high glucose (HG) for each cell line were first evaluated using the Cell Counting Kit‐8 (CCK‐8) assay. From the results, RSC96 and RAW264.7 cells were subsequently exposed to their respective optimal HG concentrations of 100 and 30 mM for 24 h to establish the injury model, with 5.5 mM glucose serving as the normal control. For certain experiment, Compound C (10 *μ*m) was added.

### 2.8. Cell Viability Assay (CCK‐8)

Cell viability was analyzed through the CCK‐8 by following manufacturer′s guidance. Absorbance was measured at 450 nm through a microplate reader.

### 2.9. ROS Detection

Intracellular ROS in RSC96 cells was measured using the fluorescent probe DCFH‐DA. Tissue ROS in sciatic nerve sections was assessed using dihydroethidium (DHE). Fluorescence images were captured under consistent settings, and mean fluorescence intensity (MFI) was quantified using ImageJ.

### 2.10. Antioxidant Enzyme Activities

Superoxide dismutase (SOD) and glutathione peroxidase (GSH‐Px) activities in sciatic nerve homogenates were measured using commercial kits and normalized to the total protein concentration.

### 2.11. TUNEL Assay

Apoptosis in sciatic nerve sections was detected using a one‐step TUNEL kit. Sections were counterstained with DAPI, and the apoptotic index was calculated as the percentage of TUNEL‐positive cells.

### 2.12. Immunofluorescence

Paraffin‐embedded sciatic nerve sections and cultured RAW264.7 cells were subjected to immunofluorescence staining through primary antibodies against Bcl‐2, p‐mTOR, iNOS, and Arg1. Secondary antibodies conjugated with Alexa Fluor 488 or 594 were used. Images were acquired through a fluorescence microscope and analyzed with ImageJ.

### 2.13. RNA Extraction and Quantitative Real‐Time PCR (qRT‐PCR)

Total RNA was extracted from RSC96 cells using a magnetic bead‐based kit. cDNA was synthesized and subjected to qPCR. Gene expression levels of *BCL2*, *EGFR*, *mTOR*, and *MMP9* were normalized to *β*‐actin and calculated using the 2^−△△CT^ method. The sequences of the primers used are included in Table S2.

### 2.14. Western Blotting

Protein extracts from sciatic nerves were detached by SDS‐PAGE, transferred to PVDF membranes, and probed with antibodies against AMPK, p‐AMPK, mTOR, p‐mTOR, Bcl‐2, and *β*‐actin. Band intensities were quantified using ImageJ.

### 2.15. Network Pharmacology

Res targets were retrieved from SwissTargetPrediction (https://swisstargetprediction.ch/), and DPN‐related genes were gathered from GeneCards (https://www.genecards.org/). Overlapping targets were analyzed for protein–protein interactions (PPI) using STRING (https://cn.string-db.org/) and visualized in Cytoscape (Version 3.9.1). Functional enrichment analysis was performed using the DAVID database (https://david.ncifcrf.gov/) [17].

### 2.16. Molecular Docking

The 3D structure of Res was obtained from PubChem (https://pubchem.ncbi.nlm.nih.gov/). Protein structures were retrieved from the UniProt database (https://www.uniprot.org/uniprotkb) and prepared using PyMOL (https://pymol.org/). Docking simulations were done through AutoDock Vina (V1.2.x), and findings were visualized with PyMOL.

### 2.17. Mendelian Randomization (MR) Analysis

Two‐sample MR was performed to evaluate the causal relationship between expression levels of the nominated target genes and DPN risk. Cis‐expression quantitative trait loci (cis‐eQTLs) for *PRKAA1* (encoding AMPK‐a1), *PRKAA2* (encoding AMPK‐a2), *MTOR*, and *BCL2* were obtained from the eQTLGen consortium (*n* = 31,684) and used as instrumental variables. Genome‐wide association study (GWAS) summary statistics for diabetic polyneuropathy were obtained from the FinnGen study (release R10; phenotype code G6_DIABET_POLYNEU). Instrument selection was restricted to genome‐wide significant cis‐eQTLs (*p* < 5 × 10^−8^) located within 1 Mb of the transcription start site. Clumping was applied at *r* ^2^ < 0.001 and a 10,000 kB window to ensure independence. The primary causal estimate was derived using the inverse‐variance weighted (IVW) method. Sensitivity analyses included the MR‐Egger regression, weighted median, and weighted mode methods. Heterogeneity was assessed using Cochran′s *Q* test, and horizontal pleiotropy was evaluated with the MR‐Egger intercept test. Results were considered direction‐consistent if the MR‐estimated causal direction matched the experimentally observed role of each target (i.e., protective for AMPK and BCL2, risk‐promoting for mTOR). The analysis was conducted using the TwoSampleMR (V0.6.6) and ieugwasr (V1.0.1) R packages.

### 2.18. Mutational Constraint Analysis at Res‐Binding Pockets

To assess the evolutionary constraint acting on the Res‐binding pocket residues identified by molecular docking, missense variant data for BCL2 (UniProt: P10415) and MTOR (UniProt: P42345) were retrieved from the Genome Aggregation Database (gnomAD V4; > 800,000 individuals). Protein domain annotations and full‐length amino acid sequences were obtained from UniProt. Binding pocket residues were defined based on the molecular docking results: 14 residues for BCL2 (F104, Y108, D111, F112, M115, V133, E136, L137, G141, G145, R146, A149, F153, V156) and 11 residues for MTOR (L2185, K2187, E2190, P2229, S2231, V2240, D2338, M2345, L2354, D2357, D2360), including residues forming hydrogen bonds and hydrophobic contacts with Res.

Variant density was calculated as the number of missense variants per residue position, separately for binding pocket and nonbinding regions of each protein. CADD (Combined Annotation Dependent Depletion) scores from gnomAD annotations were compared between binding pocket and nonbinding variants using the Wilcoxon rank‐sum test. Per‐position variant counts were plotted across the entire protein to visualize the spatial distribution of missense variants relative to the binding pocket.

An evidence convergence matrix was constructed to integrate five dimensions of evidence for each target gene: MR causal significance (−log_10_ *p* value), direction consistency with experimental findings (binary), molecular docking binding energy (|kcal/mol|/2), binding pocket evolutionary constraint (nonbinding‐to‐binding variant density ratio), and mutation functional impact (mean CADD score at binding residues/6). All scores were normalized to a 0–5 scale. All analyses were performed in R (V4.4.2) and visualized using ggplot2, ComplexHeatmap, and patchwork.

### 2.19. Statistical Analysis

Data are presented as mean ± SEM. For in vivo experiments, *n* = 8 mice in every group. For in vitro assays, experiments were independently repeated three times (*n* = 3). Repeated‐measures data were analyzed trough two‐way ANOVA with Tukey′s post hoc test; other data were contrasted through one‐way ANOVA with Tukey′s test using GraphPad Prism. Significance was set at *p* < 0.05.

## 3. Results

### 3.1. Res Alleviates Pathological Symptoms and Promotes Nerve Repair in DPN Mice

The therapeutic impacts of Res on DPN were systematically evaluated in STZ‐induced diabetic mice. Figure 1A illustrates the experimental design and grouping strategy of the animal study. During the experimental period, DPN mice exhibited significant reductions in body weight and marked increases in FBG relative to control animals (*p* < 0.001). Res treatment significantly improved both parameters in a dose‐dependent manner, with the high‐dose group (Res‐H) showing effects comparable to the ALA group (Figures 1B,D). MNCV was markedly decreased in DPN mice. The treatment of both ALA and Res reversed the MNCV levels to normal (*p* < 0.01) (Figure 1C). Functional recovery was also aided by behavioral tests. DPN mice showed decreased thermal withdrawal and mechanical paw‐withdrawal threshold, which are signs of sensory impairment. Such losses were dramatically improved with the use of Res in a dose‐dependent effect (*p* < 0.05–0.001)(Figure 1E,F). Through H&E, Masson staining and LFB staining, histological examination of sciatic nerves in the DPN group had severe swelling of axons, demyelination, and deposition of collagen (Figure 1G–I). Conversely, the abnormalities were greatly reduced with Res‐H and ALA treatment because of better myelin integrity, less axonal swelling, and less fibrosis than it was in the DPN group. The use of transmission electron microscopy (TEM) demonstrated intensive atrophy of axons and rupture of myelin sheaths in the DPN model group (Figure 1J). Res treatment, and especially at the high concentration (Res‐H) dose, significantly decreased these abnormalities, leading to axons of rounder shape and with thicker and denser myelin sheaths. To validate that Res (Res‐H) is an important ameliorative of ultrastructural injury, quantitative morphometric analyses proved that Res enhanced axonal diameter, myelin sheath thickness, and by a combination of both, optimized G‐ratio relative to the DPN model. These results suggested that Res may enhance efficiency of myelination and the integrity of the nerve fibers that are comparable to the ALA group (Figure 1K–M).The overall findings in these results suggest that Res may possess neuroprotective and repairing properties in diabetic neuropathy treatment.

Figure 1Resveratrol alleviates pathological symptoms and promotes nerve repair in DPN mice. (A) Schematic illustration of the experimental design and drug administration protocol. (B) Body weight and (D) fasting blood glucose (FBG) levels were tracked monthly throughout the experimental period. (C) Motor nerve conduction velocity (MNCV) was determined during the study endpoint. (E) Thermal withdrawal latency of the hind paw was assessed using the Hargreaves test, and (F) mechanical paw withdrawal threshold was analyzed using von Frey filaments on a monthly basis. (G) Representative hematoxylin and eosin (H&E) staining images of sciatic nerves. (H) Representative Masson’s trichrome staining images showing collagen fiber deposition (blue). (I) Representative Luxol Fast Blue (LFB) staining images displaying myelinated nerve fibers. (J) Transmission electron microscopy (TEM) images of sciatic nerves; the red boxes indicate the magnified areas. (K–M) Quantitative analysis of axonal and myelin morphology from TEM images: (K) Axon diameter, (L) myelin diameter, and (M) G‐ratio. Data are indicated as average ± SEM (n = eight mice for every group). The Statistical relevance was assessed using a two‐way ANOVA (for Panels B, D, E, and F) or one‐way ANOVA (for Panel C) then by usingTukey’s post hoc test. ∗ *p* < 0.05, ∗∗ *p* < 0.01, ∗∗∗ *p* < 0.001 vs. control group; #*p* < 0.05, ##*p* < 0.01, ###*p* < 0.001 vs. DPN group; $*p* < 0.05, $$*p* < 0.01, $$$*p* < 0.001 vs. DPN+Res‐L group.

**Figure:**
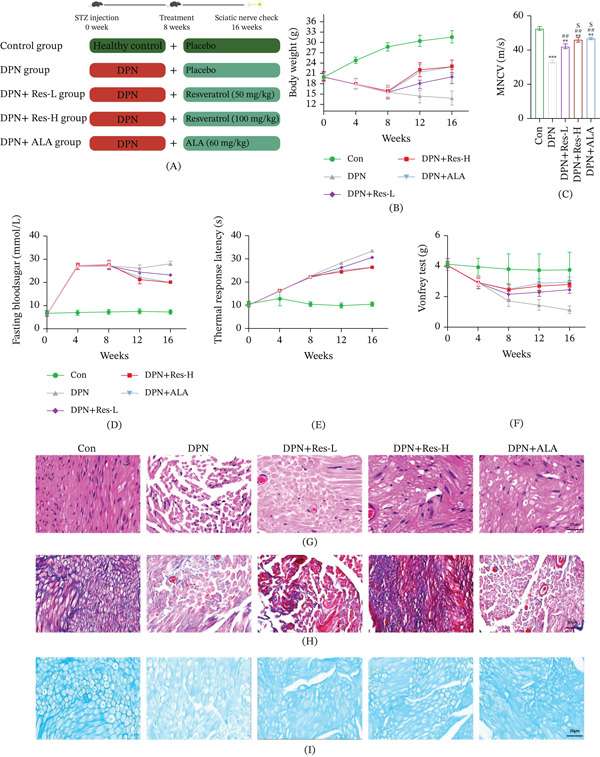
(A)

**Figure:**
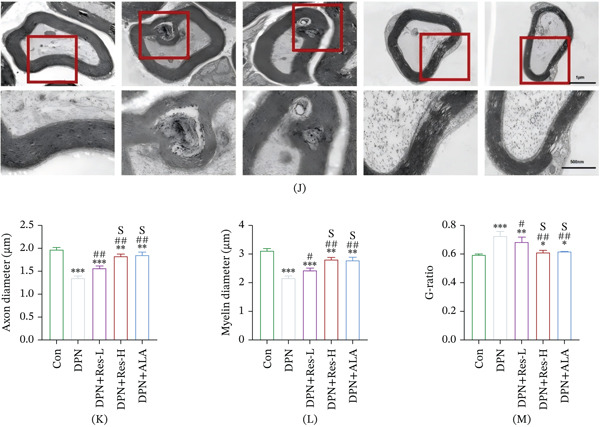
(B)

### 3.2. Network Pharmacological Analysis Identifies Potential Targets and Mechanisms of Res in DPN

**Figure 2:**
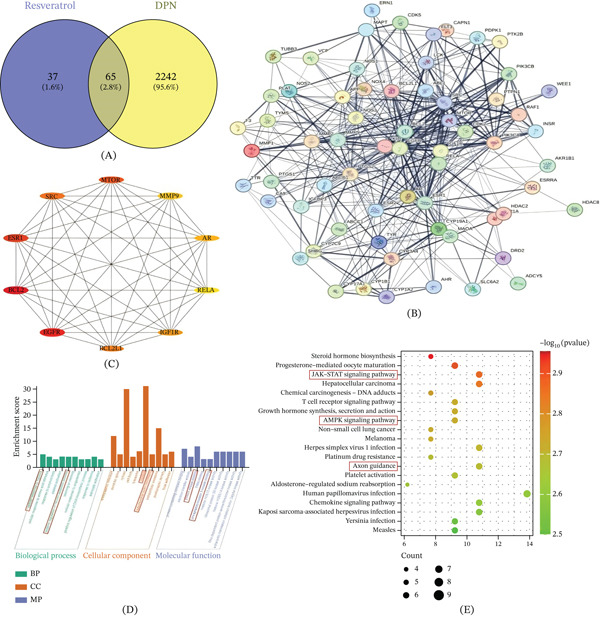
Network pharmacological analysis reveals potential targets and signaling pathways of resveratrol in DPN. (A) Venn diagram showing the convergence between predetermined targets of resveratrol and differentially expressed genes (DEGs) shown in the DPN model. The overlapping region represents the potential therapeutic targets. (B) Protein–protein interaction (PPI) network of the overlapping genes made by utilizing the STRING database. The node size shows the level of connection, with larger nodes showing more central targets. (C) Magnified view of the top 10 hub genes within the PPI network, likely representing key nodes in the resveratrol‐DPN interaction network. (D) Gene Ontology (GO) enrichment analysis of the overlapping targets, illustrating the top notably enhanced terms in biological process (BP), cellular component (CC), and molecular function (MF) categories. (E) Kyoto Encyclopedia of Genes and Genomes (KEGG) pathway enrichment analysis of the overlapping targets, with bubble color and size representing –log10 (p value) and gene count, respectively.

To recognize posible therapeutic targets of Res, we first retrieved 102 Res‐related target genes from the SwissTargetPrediction database. Meanwhile, 2307 genes connected with DPN were obtained from the GeneCards database. A Venn diagram was constructed to determine the overlapping targets, revealing 65 potential Res‐targeted genes implicated in DPN (Figure 2A, Table S3). PPI network revealed multiple highly connected nodes, showing that Res may employ its pharmacological actions by multitarget and multipathway mechanisms (Figure 2B). The top 10 hub genes, identified based on degree centrality, were primarily associated with the regulation of oxidative stress, inflammatory response, and energy metabolism. These hub genes included *MTOR*, *MMP9*, *AR*, *RELA*, etc. (Figure 2C). Gene Ontology (GO) enrichment analysis showed that, in the biological process category, the differentially expressed genes were mainly enriched in cellular response to hypoxia and positive regulation of neuron apoptotic process, suggesting a close association with hypoxic stress and neuronal cell death. In the cellular component category, significantly enriched terms included nucleus and mitochondrial outer membrane, indicating that many of the encoded proteins are localized in the nucleus and mitochondrial membrane‐associated structures. For the molecular function category, the genes were predominantly enriched in protein kinase binding, FMN binding and phosphatidylinositol 3‐kinase binding, highlighting the involvement of kinase‐mediated signaling and redox‐related functions in this context (Figure 2D). KEGG pathway enrichment analysis showed that the differentially expressed genes were notably enriched in a number of canonical pathways, such as the JAK‐STAT signaling pathway, AMPK signaling pathway, and axon guidance (Figure 2E). Among them, the JAK‐STAT and AMPK signaling pathways showed the highest enrichment significance, suggesting a critical involvement of inflammatory/immune signaling and energy metabolism reprogramming in DPN. These findings suggest that Res may exert therapeutic impacts in DPN through the regulation of key genes involved in oxidative stress, inflammation, and energy metabolism, particularly via the JAK‐STAT and AMPK signaling pathways.

### 3.3. MR and Mutational Constraint Analysis Support the Causal Involvement of Res Targets in DPN

**Figure 3:**
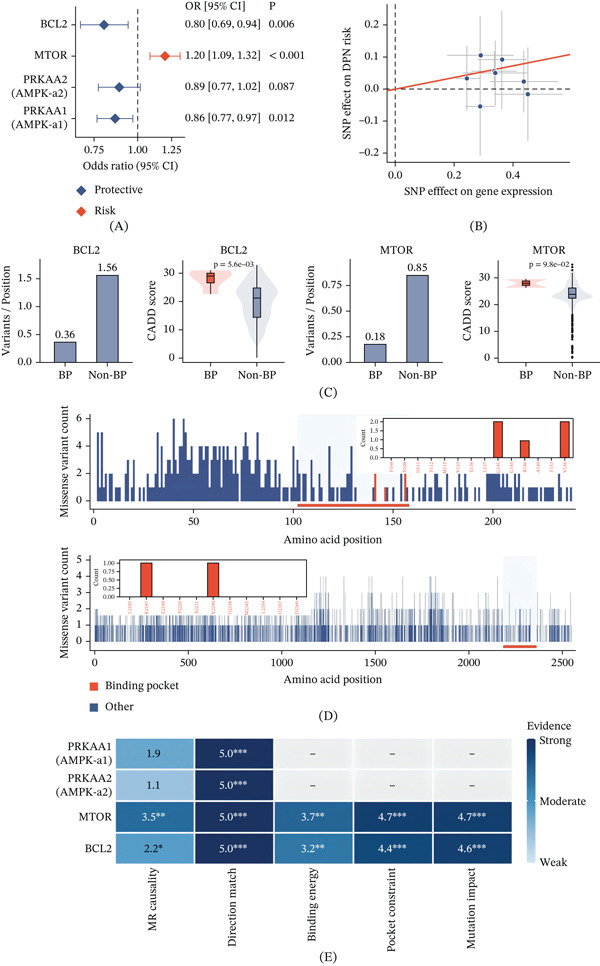
Mendelian randomization and mutational constraint analysis validate resveratrol targets in DPN. (A) Forest plot of two‐sample MR results (IVW method) for genetically predicted expression of four target genes and DPN risk. Blue diamonds indicate protective effects (OR < 1); red diamonds indicate risk effects (OR > 1). OR with 95% CI and p values are listed on the right. (B) MR scatter plot for MTOR, showing SNP‐level effects on gene expression (x‐axis) versus DPN risk (y‐axis); the red line is the IVW regression slope. (C) Evolutionary constraint at resveratrol binding pockets. Left pair: variant density (missense variants per residue position) in binding pocket (BP) versus nonbinding (Non‐BP) regions for BCL2 and MTOR. Right pair: CADD score distributions for variants at BP versus Non‐BP positions; Wilcoxon test p values are shown. (D) Per‐position missense variant counts across the full‐length BCL2 (upper) and MTOR (lower) proteins (gnomAD v4). Blue bars, nonbinding positions; red bars, binding pocket positions. Cyan shading and red horizontal bars mark the binding pocket region. Insets show variant counts at individual binding residues. (E) Evidence convergence matrix integrating MR causality, direction match with experimental data, molecular docking binding energy, pocket evolutionary constraint, and mutation functional impact for each target gene. Cell values represent evidence strength scores (0–5 scale); asterisks indicate significance (∗ *p* < 0.05, ∗∗ *p* < 0.01, ∗∗∗ *p* < 0.001 or equivalent); dashes indicate data not available.

To assess whether the network pharmacology‐nominated targets have a causal relationship with DPN at the human genetic level, we performed two‐sample MR using cis‐eQTLs for PRKAA1, PRKAA2, MTOR, and BCL2 as instrumental variables, which were rigorously selected from authoritative public eQTL datasets following standard cis‐regulatory and genome‐wide significance criteria, and DPN‐related GWAS summary statistics as the outcome. The IVW method indicated that genetically predicted higher expression of MTOR was associated with increased DPN risk (OR = 1.20; 95% CI 1.09–1.32; *p* < 0.001), whereas higher BCL2 expression was associated with reduced risk (OR = 0.80; 95% CI 0.69–0.94; *p* = 0.006) (Figure 3A). PRKAA1 showed a similar protective trend (OR = 0.86; 95% CI 0.77–0.97; *p* = 0.012). PRKAA2 did not reach significance (*p* = 0.087). The causal directions from MR were fully concordant with the protective (AMPK/BCL2) and pathogenic (mTOR) roles observed in our in vivo and in vitro experiments. The MR scatter plot for MTOR confirmed a positive slope across individual SNP instruments without evidence of directional pleiotropy (Figure 3B).

We next examined whether the Res binding pockets identified by molecular docking are subject to evolutionary constraint in the human population. From gnomAD V4 (> 800,000 individuals), we retrieved 355 missense variants for BCL2 and 2150 for MTOR, and compared variant density at binding pocket residues versus the rest of each protein. For BCL2, binding pocket positions carried 0.36 variants per residue compared with 1.56 for nonbinding positions, a 4.3‐fold depletion (Figure 3C, left). For MTOR, the ratio was 0.18 versus 0.85, a 4.7‐fold depletion (Figure 3C, right). Furthermore, the few missense variants that did occur at binding pocket positions had significantly higher CADD pathogenicity scores than variants elsewhere in BCL2 (Wilcoxon *p* = 5.6 × 10^−3^) (Figure 3C), indicating that mutations at these sites are under strong negative selection.

To visualize this depletion in genomic context, we plotted per‐position missense variant counts along the entire protein sequence for BCL2 and MTOR. In both proteins, the Res binding pocket region showed a conspicuous gap in variant density compared with adjacent regions (Figure 3D). Zoomed insets confirmed that of the 14 BCL2 binding residues (F104, Y108, D111, F112, M115, V133, E136, L137, G141, G145, R146, A149, F153, V156), 12 had zero observed missense variants, and only G141 and G145 had one or two. The 11 MTOR binding residues (L2185, K2187, E2190, P2229, S2231, V2240, D2338, M2345, L2354, D2357, D2360) were similarly depleted, with only K2187 and V2240 carrying a single variant each.

Finally, we integrated MR causality, direction consistency, binding affinity (from molecular docking), pocket constraint, and mutation impact into a convergence matrix across all four target genes (Figure 3E). MTOR and BCL2 received strong evidence scores (3.2–4.7 out of 5.0) across all five dimensions. PRKAA1 and PRKAA2 showed moderate MR evidence and full direction consistency, but lacked direct binding and constraint data because they were not among the docked targets. These orthogonal lines of evidence collectively support the biological plausibility of the AMPK/mTOR/Bcl‐2 axis as a mechanistic framework for Res′ anti‐DPN effects.

In the present study, only missense variants were included in the mutation constraint analysis. Nonsense and frameshift mutations are extremely rare in the general population and lack sufficient variant data in the gnomAD database; thus, these mutation types were not analyzed. Future studies can perform stratified mutation constraint and association analyses in East Asian populations to improve the ethnic representativeness and clinical translational potential of our findings.

### 3.4. Molecular Docking Analysis and qPCR Validation Reveal Res′ Interaction With and Regulation of Key Targets

**Figure 4:**
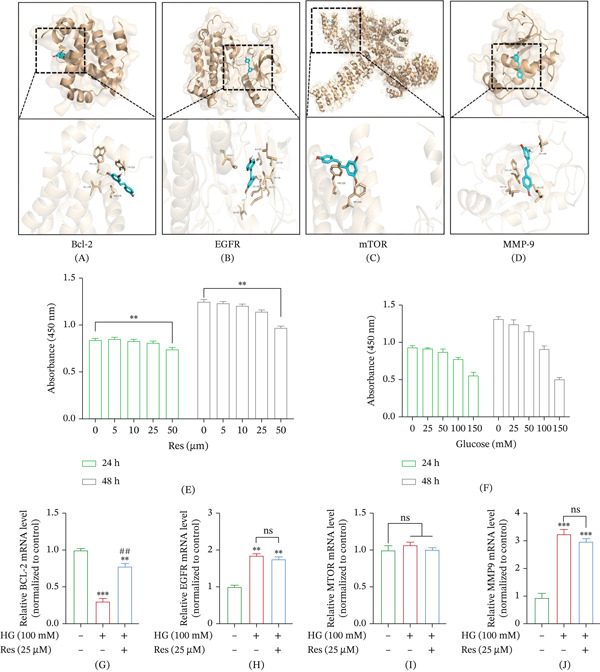
Molecular docking analysis and qPCR validation reveal resveratrol’s interaction with and regulatory effects on key targets. (A–D) Molecular docking models predicting the binding poses of resveratrol (cyan) with the putative target proteins (A) Bcl‐2, (B) EGFR, (C) mTOR, and (D) MMP‐9. The enlarged views show major interplay (e.g., hydrogen bonds and hydrophobic contacts) within the binding pockets; proteins are shown in brown and key residues are highlighted. (E) Dose screening of resveratrol (Res) based on cell viability (CCK‐8, absorbance at 450 nm) at 24 hours and 48 hours. (F) High‐glucose (HG) modeling concentration screening based on cell viability (CCK‐8, absorbance at 450 nm) at 24 h and 48 h. (G–J) Relative mRNA expression levels of (G) Bcl‐2, (H) EGFR, (I) mTOR, and (J) MMP‐9 in sciatic nerve tissues from different experimental groups, determined using quantitative real‐time PCR (qRT‐PCR). Data are indicated as average ± SEM; for in vitro assays (E–F), n = 3 independent experiments, and for in vivo qRT‐PCR analyses (G–J), n = 8 mice per group. Statistical evaluation was performed through one‐way ANOVA then by Tukey’s post hoc test.∗ *p* < 0.05, ∗∗ *p* < 0.01, ∗∗∗ *p* < 0.001 vs. control; #*p* < 0.05, ##*p* < 0.01, ###*p* < 0.001 vs. HG; ns shows negligible variation (*p* > 0.05).

To further evaluate the interactions between Res and key targets, molecular docking was performed for proteins encoded by the top 10 candidate genes, and the best binding energies (kcal/mol) were compared (Table S4). Res showed the strongest predicted binding to MMP9 (−9.3 kcal/mol), followed by mTOR (−7.4 kcal/mol), EGFR (−7.0 kcal/mol), and Bcl‐2 (−6.4 kcal/mol). These findings indicate that Res can have a direct interaction with various targets, which gives a structural rationale to its possible involvement in the inflammation‐, apoptosis‐, and proliferation‐related mechanisms. Representative docking of the four proteins is shown in Figure 4A–D.

To test these computational predictions, we now investigated the actions of Res with HG‐induced Schwann‐cell model DPN in RSC96 cells. CCK‐8 indicated that Res induced cytotoxicity at 50 *μ*m, but no apparent cytotoxicity was detected at concentrations lower than 25 *μ*m (Figure 4E). Thus, 25 mM was chosen further experiments. With the same assay, we established that 100 mM glucose effectively decreased the cell viability, which was selected as the HG injury inducing condition (Figure 4F).

We then determined the effect of HG and Res intervention on predictive targets transcription by means of qRT‐PCR in which HG stimulation was significantly suppressing *BCL2* mRNA expression and was reversed by Res intervention (Figure 4G). Conversely, HG and Res did not have significant effects on *MTOR* mRNA levels (Figure 4I). Under HG conditions, *EGFR* was found to be increased and Res had no effect on the expressional level of *EGFR* as compared to the level in the HG group (Figure 4H). Equally, the treatment of HG enhanced the expression of *MMP9*, which was not mitigated by Res in comparison to HG (Figure 4J). Notably, *MMP9* showed strong binding affinity in molecular docking but no significant change at the mRNA level under Res treatment. This discrepancy suggests that Res may regulate *MMP9* through posttranslational modifications or PPI rather than transcriptional regulation.

Combined, these data suggest that Res may have therapeutic effects in part by direct action on core targets such as Bcl‐2 and this reversal of the HG‐induced transcriptional dysregulation, and not by a widespread change in the mRNA expression of all the predicted docking targets.

### 3.5. Res Ameliorates DPN by Attenuating Oxidative Stress and Neuronal Apoptosis

**Figure 5:**
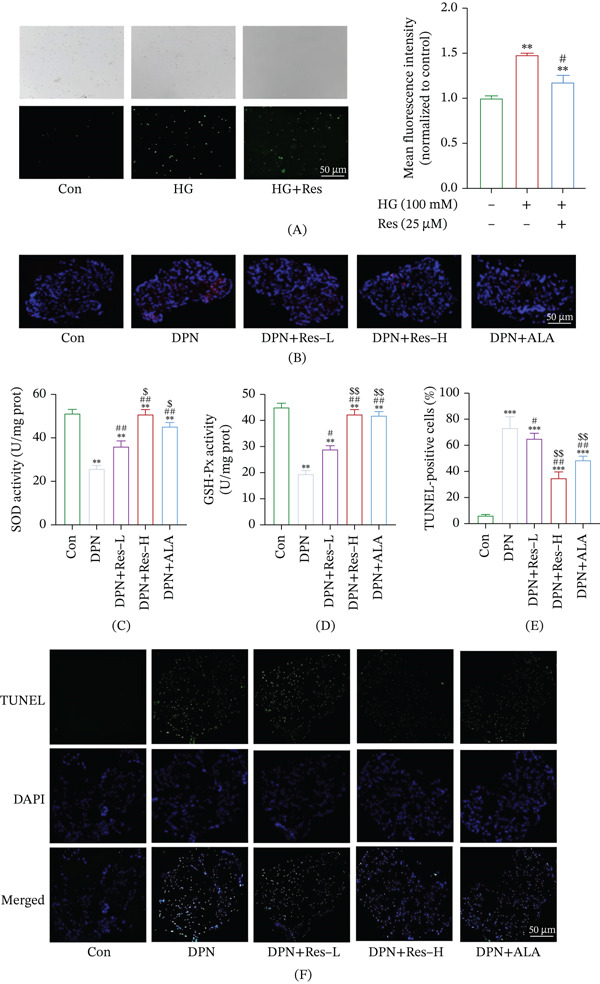
Resveratrol mitigates oxidative stress and neuronal apoptosis in DPN models. (A) Left panel: Representative bright‐field (top) and DCFH‐DA fluorescence (bottom) images of RSC96 Schwann cells. From left to right: control group (Con), high glucose group(HG, 100mM), and high glucose with resveratrol treatment group (HG+Res). Green fluorescence indicates intracellular ROS levels. Right panel:Quantitative analysis of the mean DCF fluorescence intensity. (B)Representative dihydroethidium (DHE) staining images of sciatic nerve sections from the Control, DPN, DPN+Res‐L, DPN+Res‐H and DPN+ALA groups. Red fluorescence indicates superoxide anion levels. (C, D) Quantitative analysis of antioxidant enzyme activities in sciatic nerve homogenates: (C) superoxide dismutase (SOD) and (D) glutathione peroxidase (GSH‐Px). (E) Apoptotic index, represented by the percentage of TUNEL‐positive cells, in sciatic nerves across different groups. (F) Representative TUNEL staining images of sciatic nerve sections. Apoptotic nuclei are stained green (TUNEL), and all nuclei are counterstained blue (DAPI). The merged images show co‐localization. Data are indicated as mean ± SEM; for in vitro assays (A), n = 3 independent experiments, and for in vivo analyses (B–F), n = 8 mice for all groups. Statistical relevance was evaluated through one‐way ANOVA then by Tukey’s post hoc test. ∗ *p* < 0.05, ∗∗ *p* < 0.01, ∗∗∗ *p* < 0.001 vs. control group; #*p* < 0.05, ##*p* < 0.01, ###*p* < 0.001 vs. HG OR DPN group; $*p* < 0.05, $$*p* < 0.01, $$$*p* < 0.001 vs. DPN+Res‐L group.

To ascertain the neuroprotective potential of Res in connection to mitigated oxidative damage and subsequent cellular degradation, we conducted complementary in vitro and in vivo research to study the ROS production, endogenous antioxidants capacity and cellular degradation or apoptosis of Schwann cells under diabetic conditions. The HG in RSC96 Schwann cells significantly enhanced intracellular ROS when assessed by a significant rise in the DCFH‐DA fluorescence. Remarkably, the treatment with Res caused a reduction in the fluorescence of DCFH‐DA by far, which also revealed that the HG‐mediated accumulation of ROS was also suppressed (Figure 5A). These findings show that Res directly influences suppression of the oxidative stress at cellular level in a hyperglycemic microenvironment.

In accordance with the in vitro results, in vivo DHE staining revealed strong overproduction of ROS in the cryogenic nerves that are sciatic in DPN mice as opposed to controls. The application of Res significantly reduced the DHE fluorescence signal, and the reduced signal developed a distinct dose‐dependent trend, with the high‐dose group reporting a stronger signal (Figure 5B). The consistency between cellular and tissue level analysis here reinforces the argument that Res is protective against oxidative stress in peripheral nerves in the development of DPN.

In order to further assess the activity of Res in repairing impaired antioxidant defenses by diabetes, we quantified the activity of major antioxidant enzymes. The activities of SOD and GSH‐Px were biochemically determined and indicated reduced endogenous production of antioxidant functions and a lack of redox homeostasis in DPN mice (Figure 5C, 5D). Notably, SOD and GSH‐Px activity was successfully replenished to control levels by the Res treatment, which indicates that Res does not just reduce the accumulation of ROS but also improves the intrinsic antioxidant mechanisms to fight the oxidative damage.

Since the overabundance of oxidative stress is a key cause of diabetic neuropathy‐induced Schwann‐cell dysfunction and apoptosis, we subsequently analyzed the apoptotic alterations in the peripheral nerve tissue. Apoptosis was identified by TUNEL staining in the sciatic nerves of mice with DPN and the number of TUNEL‐positive nuclei was significantly higher in contrast with controls. The percentage of TUNEL positive cells was significantly less with Res intervention which is a strong indication of anti‐apoptotics in diabetic peripheral nerves (Figure 5E, 5F). Since myelin survival relies on Schwann cells along with the maintenance of axons and the overall health of nerves, the inhibition of apoptosis by Res may possibly help maintain the beauty of peripheral nerves and their functions under hyperglycemic conditions.

Together, these data indicate that Res has strong antioxidative and anti‐apoptotic impacts on DPN. Under persistent hyperglycemic conditions, Res may preserve Schwann‐cell integrity, thereby inhibiting both neural degeneration and the progress of DPN through reducing ROS overload of metabolites, restoring antioxidant enzyme activity (SOD and GSH‐Px), and inhibiting Schwann‐cell apoptosis.

### 3.6. Res Activates the AMPK/mTOR/Bcl‐2 Signaling Axis to Confer Neuroprotection in DPN

To further explain the molecular foundation of the antioxidative and anti‐apoptotic actions of Res, we targeted the AMPK/mTOR/Bcl‐2 signaling pathway as a prominent controller of energy homeostasis, stress response, and survival in cells. AMPK suppression and mTOR activation have also been associated to hyperglycemia‐impaired metabolic functions and Schwann‐cell damage, which eventually leads to peripheral nerve degeneration in DPN [18, 19].

**Figure 6:**
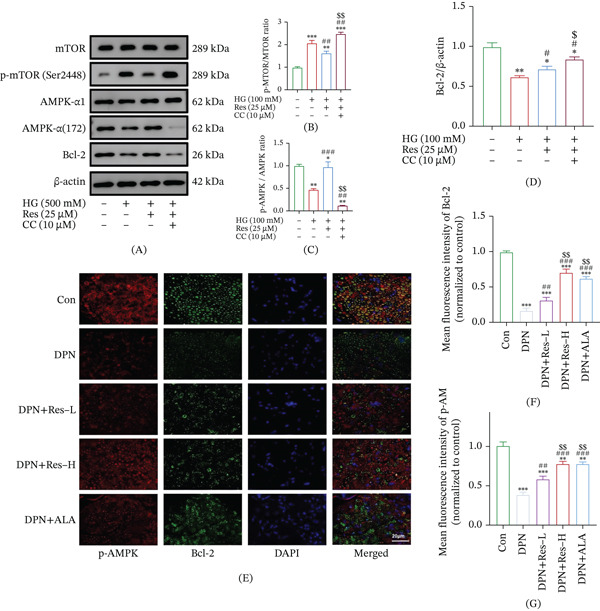
Resveratrol activates the AMPK/mTOR/Bcl‐2 signaling axis to confer neuroprotection in DPN. (A) Representative Western blot images indicating the protein expression levels of mTOR, phospho‐mTOR (Ser2448), AMPK‐*α*1, phospho‐AMPK‐*α* (Thr172), Bcl‐2, and *β*‐actin in sciatic nerve tissues from different experimental groups: Control, DPN, DPN+Res‐L, DPN+Res‐H, and DPN+CC. (B–E) Quantitative analysis of the protein expression levels normalized to *β*‐actin or the ratio of phosphorylated to total protein: (B) Bcl‐2/*β*‐actin, (C) p‐mTOR/mTOR, (D) p‐AMPK/AMPK. (E) Representative immunofluorescence staining images of sciatic nerve parts from the Control, DPN, DPN+Res‐L, DPN+Res‐H, and DPN+ALA groups. Sections were stained for Bcl‐2 (green) and p‐AMPK (red). Nuclei were counterstained with DAPI (blue). Combined images indicate the co‐localization of signals. (F–G)Quantitative evaluation of the average fluorescence intensity (MFI) for (F) Bcl‐2 and (G) p‐AMPK from the images in panel E. Data in B–D and F–G are indicated as average ± SEM (n = eight mice for all groups). Statistical importance was evaluated through one‐way ANOVA then using Tukey′s post‐hoc test. ∗ *p* < 0.05, ∗∗ *p* < 0.01, ∗∗∗ *p* < 0.001 vs. control group; #*p* < 0.05, ##*p* < 0.01, ###*p* < 0.001 vs. DPN group; $*p* < 0.05, $$*p* < 0.01, $$$*p* < 0.001 vs. DPN+Res‐L group.

Western blotting in the HG‐stimulated RSC96 cells revealed that there was a strong inhibition of AMPK activation (reduced p‐AMPK, Thr172) with significant enhancement of mTOR activation (p‐mTOR, Ser2448) (Figure 6A, 6B, and 6C). In line with increased apoptotic vulnerability in the hyperglycemic stressor, the expression of the Bcl‐2 protein was also significantly reduced in comparison to controls (Figures 6A, 6B, 6C, and 6D). It is worth noting that the treatment with Res strongly reversed these HG‐induced signaling defects: p‐AMPK levels returned to normal, p‐mTOR levels decreased to basal levels, and Bcl‐2 levels were significantly elevated (Figures 6A, 6B, 6C, and 6D). Res significantly upregulated Bcl‐2 protein expression but had a milder effect on mRNA level, indicating that Res regulates Bcl‐2 mainly at the protein level. This evidence suggests that Res restores the HG‐induced switch in energy‐conserving and maladaptive mTOR‐dominant signaling phenotype.

These biochemical results were supported independently using immunofluorescence staining. The DPN group showed advance fluorescence emission of the p‐AMPK and Bcl‐2 in sciatic nerve tissues, and it can be observed that the staining intensity of both markers was enhanced by Res administration (Figure 6E). In sciatic nerve tissues, the DPN group exhibited reduced fluorescence intensity of phosphorylated AMPK (p‐AMPK) and Bcl‐2 (Figures 6F, 6G), which were significantly restored by Res treatment. These results indicate the regional upregulation of p‐AMPK and its positive spatial correlation with Bcl‐2 in nerve tissue. Combined, these data demonstrate that Res increases the phosphorylation level of AMPK and inhibits mTOR hyperactivation, thereby restoring the anti‐apoptotic function mediated by Bcl‐2.

These biochemical results were supported independently using immunofluorescence staining. The DPN group showed advance fluorescence emission of the p‐AMPK and Bcl‐2 in sciatic nerve tissues, and it can be observed that the staining intensity of both markers was enhanced by Res administration (Figure 6E). In sciatic nerve tissues, the DPN group exhibited reduced fluorescence intensity of phosphorylated AMPK (p‐AMPK) and Bcl‐2 (Figures 6F, 6G), which were significantly restored by Res treatment. These results indicate the regional upregulation of p‐AMPK and its positive spatial correlation with Bcl‐2 in nerve tissue. Combined, these data demonstrate that Res increases the phosphorylation level of AMPK and inhibits mTOR hyperactivation, thereby restoring the anti‐apoptotic function mediated by Bcl‐2.

All of these findings are consistent with a model where Res restores hyperglycemia‐induced Schwann‐cell dysfunction and peripheral nerve injury at the very least by reprogramming AMPK/mTOR/Bcl‐2 signaling. It is possible that activation of AMPK and simultaneous inhibition of mTOR enhances cellular energy homeostasis and tolerance to stress, and an increase in Bcl‐2 reinforces mitochondrial structure and suppresses apoptosis. This mechanistic axis may offer a reasonable signaling pathway connecting Res to the obtained neuroprotective effects in DPN.

### 3.7. Res Mitigates Neuroinflammation and Promotes M2 Macrophage Polarization via the AMPK/Bcl‐2 Pathway

**Figure 7:**
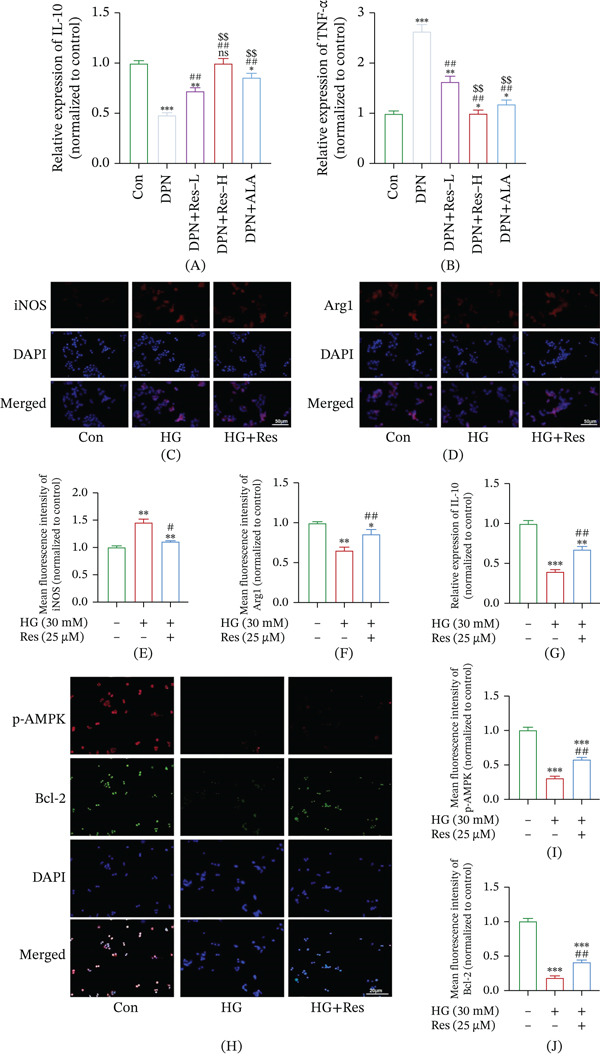
Resveratrol ameliorates diabetic peripheral neuropathy by attenuating oxidative stress and neuronal apoptosis. (A–B) Representative ELISA results: Showing the relative expression levels (normalized to the control group) of the anti‐inflammatory cytokine IL‐10 (A) and pro‐inflammatory cytokine TNF‐*α* (B) in sciatic nerve homogenates from the control, DPN, DPN+Res‐L, DPN+Res‐H, and DPN+ALA. (C–D) Representative immunofluorescence images of RAW264.7 macrophages under HG conditions: (C) displays the expression of iNOS; (D) displays the expression of Arg1. Nuclei were counterstained with DAP, and “Merge” represents the overlay of fluorescent signals. Groups include the control, HG and HG+Res groups. (E–F) Quantitative analysis of mean fluorescence intensity (MFI) from immunofluorescence: (E) corresponds to the MFI of iNOS; (F) corresponds to the MFI of Arg1. (G) ELISA quantification of IL‐10 levels in culture supernatants, with groupings consistent with (E–F). (H) Representative immunofluorescence co‐staining images of RAW264.7 macrophages under HG conditions: Detecting p‐AMPK and Bcl‐2. Nuclei were counterstained with DAPI and “Merge” represents the overlay of fluorescent signals. (I–J) Quantitative analysis of mean fluorescence intensity (MFI) from immunofluorescence: (I) corresponds to the MFI of p‐AMPK; (J) corresponds to the MFI of Bcl‐2. Groupings are consistent with (E–F). Data are indicated as average ± SEM; for in vivo ELISA analyses (A–B), n = 8 mice for each of the groups, and for in vitro assays (C–J), n = 3 independent experiments. Statistical relevance was evaluated through one‐way ANOVA then the Tukey’s post hoc test. ∗ *p* < 0.05, ∗∗ *p* < 0.01, ∗∗∗ *p* < 0.001 vs. control group; #*p* < 0.05, ##*p* < 0.01, ###*p* < 0.001 vs. HG OR DPN group; $*p* < 0.05, $$*p* < 0.01, $$$*p* < 0.001 vs. DPN+Res‐L group.

To clarify the anti‐inflammatory effect of Res in DPN, we initially examined the inflammatory cytokines in the sciatic nerve tissues. ELISA findings showed that there was an evident inflammatory imbalance in DPN mice with a marked decrease in the anti‐inflammatory cytokine IL‐10 (Figure 7A), and a subsequent increase in the pro‐inflammatory cytokine TNF‐*α* (Figure 7B). Treatment with Res immensely elevated IL‐10 and inhibited TNF‐*α*; it produces a general tendency in cytokine milieu restoration, a result that indicates attenuation of neuroinflammation in vivo.

To identify the possibility of Res in mediating macrophage‐based inflammation, we set a model of microphages being at the HG condition understimulated and determined the polarization of the macrophage. Before mechanistic assays, viability screening of CCK‐8 was done in order to establish experimental conditions. In agreement with the RSC96 model, 30 mM glucose induced the GH‐established cellular stress/injury into RAW264.7 cells and was chosen as the modeling concentration. Simultaneously, dose screening of Res revealed that 25 *μ*m did not exhibit any noticeable cytotoxicity and thus was used in further in vitro study (Figures S1 and S2).

Immunofluorescence analysis provided evidence that HG stimulation of the pro‐inflammatory M1‐like phenotype was observed via significant enhancement of the iNOS expression (Figure 7C and E). iNOS positivity decreased greatly under HG conditions with treatment with Res, implying an inhibition of M1 polarization. In turn, HG exposure minimized the expression of the anti‐inflammatory M2 representative marker, Arg1, (Figure 7D,F), and Res effectively restored the expression of Arg1, suggesting an alteration of the state toward a repairing M2‐like state. This functional phenotypic reprogramming was also accompanied by an addition of IL‐10 to the culture supernatant following Res treatment (Figure 7G), and this further supports an altered anti‐inflammatory macrophage response.

To investigate the signaling basis of this immunomodulatory action, we compared AMPK/Bcl‐2 axis activation of macrophages. Co‐immunofluorescence staining, the HG greatly minimized the signal of p‐AMPK and Bcl‐2 in the RAW264.7 cells but Res enhanced the fluorescence intensity of p‐AMPK (Figure 7H,I) and Bcl‐2 (Figure 7H,J) significantly under HG conditions. These findings imply that Res can perform energy‐sensing and pro‐survival signaling interventions in macrophages and temper HG‐activated inflammatory reactions.

**Figure 8:**
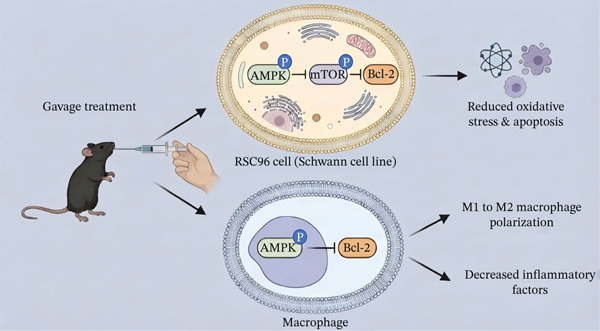
The mechanism of resveratrol on RSC96 cell and macrophage.

Together, the results show that Res may suppress DPN‐related neuroinflammation in a dual response mechanism; balancing the peripheral nerve cytokine condition and driving macrophages toward an anti‐inflammatory/repairing M2‐like phenotype. This macrophage reprogramming is probably mediated, at least in part, by AMPK/Bcl‐2 signaling pathway activation [20, 21] (Figure 8).

## 4. Discussion

DPN is one of the most common, and highly heterogeneous complications of diabetes, which is highly morbid and prevents quality of life. Although its pathogenesis, including energy‐metabolic imbalance, oxidative stress, sterile inflammation, degrading neurotrophic support, and myelin/extracellular matrix remodeling [22] has gained a better understanding, there are still no disease‐modifying therapies. The existing clinical treatment of DPN is mainly based on the glycemic control and analgesia, which provide limited improvement and do not prevent the progression of nerve damage. This highlights the great urgency to find new and more effective approach to treating the various pathways of DPN.

In this regard, network‐directed, multitarget natural products have now become potentially useful therapeutic options [23–25]. Our research indicates the possibility of Res as a treatment of DPN. Using network pharmacology in coordinate with in vivo and in vitro tests we reveal that Res can effectively enhance the mechanical thresholds, thermal latency, and the conduction of motor nerve in DPN mice. In addition, Res prevents axonal degeneration, collagen deposition, and myelin injury, which are in line with earlier observations about its neuroprotective action in the DPN models. The findings may show that the multitarget impact of Res may provide a relatively viable solution to treat DPN by effectively engaging the multifaceted pathophysiological pathways, which lead to disease development.

We found 65 potential Res targets at the network level, and the hubs identified include *BCL2*, *MTOR*, *MMP9*, and *EGFR*, which are closely associated with mitochondrial apoptosis, metabolic growth, and perineural matrix remodeling/fibrosis, respectively [26–28]. KEGG enrichment suggested AMPK as a key upstream regulator. AMPK can suppress pathological mTOR signaling and reduce oxidative and inflammatory damage by restoring metabolic homeostasis [29]. These findings indicate disrupted energy metabolism in Schwann cells contributes to DPN pathogenesis [30–32]. Multitarget agents that regulate metabolic and inflammatory pathways represent a promising strategy for DPN treatment [33]. Moreover, our mutation constraint analysis further revealed that the Res‐binding residues in *MTOR* and *BCL2* exhibited strong evolutionary conservation and extremely low missense variant density, suggesting these binding pockets are structurally stable and functionally indispensable. These findings enhance the reliability and druggability of *MTOR* and *BCL2* as therapeutic targets for DPN.

Mechanistic studies indicated that Res is a relative cytoprotective orchestrator that suppresses AMPK/mTOR/Bcl‐2 axis. A clear signature pattern, which indicated high p‐AMPK levels, low levels of p‐mTOR, and consequently the increase of Bcl‐2, supported this. These alterations led to better redox homeostasis (greater SOD/GSH‐Px levels, lower level of ROS) and less apoptotic cell mortality (less TUNEL‐positive cells). Markedly, the effects were canceled by AMPK inhibitor Compound C, which specifies the reliance on AMPK. Nevertheless, the off‐target effects of Compound C cause future researchers to consider using genetic loss‐of‐function technology or orthogonal pharmacological technology to reinforce the causal association [34]. This all points to one important integration process by which metabolic signaling is bounded by the regulation of autophagy and apoptosis. This model is further justified by literature on AMPK‐mTOR crosstalk and Bcl‐2‐Beclin 1 interactions which connects autophagy and apoptosis [35].

An immunoinflammatory biomarker Res has shown the ability to regulate immune response within DPN through the polarization of the macrophages between the harmful M1 phenotype and the repairing M2 phenotype. This change is important in alleviating neuroinflammation of DPN. The effect of Res to reduce M1 and induce M2 polarization is consistent with other studies where Res was proven to have anti‐inflammatory effects in several disease models [36, 37]. These data substantiate the premise that the AMPK/Bcl‐2 signaling cascade activation is the center of action of the immunoregulatory effects of Res. In addition, the activation of the AMPK/Bcl‐2 signaling pathway seems to underlie the immunomodulatory effects of Res. Res can possibly induce cell survival and decrease inflammation by increasing p‐AMPK and Bcl‐2 expression, and a further shift of the immune response to an anti‐inflammatory state. These findings indicate the multitargeting effect of Res, which may pose a promising treatment of DPN. But further study is required to explain the more comprehensive systemic outcome and clinical relevance of Res in the treatment of the DPN‐associated neuroinflammation.

Concerning upstream Res activation of AMPK, previous studies have proposed a SIRT1/LKB1/AMPK positive‐feedback loop, in which Res increases NAD+ to activate SIRT1, facilitates LKB1/AMPK signaling, and increases mitochondrial biogenesis and mitochondrial redox homeostasis. Nevertheless, in some circumstances, AMPK may be activated in the absence of SIRT1 [38, 39]. According to our data, experimenting with testing the need of SIRT1 with EX‐527 or SIRT1 knockdown would help shed some light on this axis.

NDPN is not a neuronal lesion alone but a systemic condition of Schwann cells, axons, microvasculature and even immunity [1]. Based on our histological, metabolic, readouts, Res restores the homeostasis of energy‐oxidative stress‐apoptosis‐inflammation continuum. This is in tandem with focused metabolomics analysis of energetic disturbances in DPN and will justify the viability of energy‐reprogramming treatments. Clinically, Res has similar antioxidant, antiallodynic properties like *α*‐lipoic acid (ALA), but it has a wider multitarget action. Scaffolding dose‐exposure relationships, rational combination failures (e.g. with ALA or metabolic modulators) and the approach to formulation will be highly beneficial in translation.

Nevertheless, our research has some limitations. First, our MR analysis was based only on FinnGen GWAS data, which may introduce potential population stratification bias and limit generalizability to other ethnic groups. Second, the STZ‐induced Type 1 diabetic mouse model cannot fully reflect the pathophysiological characteristics of human Type 2 diabetes‐related DPN, and interspecies differences should be noted. Third, Res exhibits low oral bioavailability, which may restrict its clinical efficacy and requires further optimization. Fourth, other than Schwann cells and macrophages, there is no clear evidence on how sensory neurons, endothelial cells and pericytes contribute to the neuroprotective effects of Res. Spatial transcriptomics and single‐cell sequencing might aid the mapping of intercellular metabolic‐inflammatory crosstalk. Furthermore, considering the off‐target effects of Compound C [34], and genetic AMPK loss‐of‐function models and multi‐node readouts (e.g., ULK1 phosphorylation, autophagic flux) are to be included so that causal inference is enhanced. For future clinical translation, preclinical pharmacokinetic studies (including absorption, distribution, metabolism, and excretion) are warranted to evaluate the in vivo behavior of Res. Furthermore, formulation strategies such as nanoparticle delivery, prodrug modification, or sustained‐release systems may be applied to improve its bioavailability, tissue targeting, and therapeutic efficacy in DPN.

## 5. Conclusion

In conclusion, Res alleviates DPN by attenuating oxidative stress, neuronal apoptosis, and neuroinflammation, which is mediated, at least in part, through regulation of the AMPK/mTOR/Bcl‐2 signaling axis and macrophage polarization. Mutation constraint analysis confirms the high evolutionary conservation and druggability of the key target binding pockets. These findings provide integrated genetic, structural, and experimental evidence supporting Res as a promising multitarget candidate for DPN treatment.

**Nomenclature**
Res: Resveratrol
DPN: diabetic peripheral neuropathy
AMPK: AMP‐activated Protein Kinase
mTOR: Mammalian Target of Rapamycin
Bcl‐2: B‐cell lymphoma 2
ALA: *α*‐lipoic acid
CMC‐Na: carboxymethyl cellulose sodium
FBG: fasting blood glucose
MNCV: motor nerve conduction velocity
H&E: hematoxylin and eosin
LFB: Luxol Fast Blue
TEM: transmission electron microscopy
HG: high glucose
ROS: reactive oxygen species
DHE: dihydroethidium
SOD: superoxide dismutase
GSH‐Px: glutathione peroxidase
CC: Compound C
qRT‐PCR: quantitative real‐time PCR
ELISA: Enzyme‐Linked Immunosorbent Assay
PVDF: polyvinylidene fluoride
PPI: protein‐protein interaction
GO: gene ontology
KEGG: Kyoto Encyclopedia of Genes and Genomes
MFI: mean fluorescence intensity
DCFH‐DA 2 ^′^,7 ^′^
: Dichlorodihydrofluorescein diacetate

## Author Contributions

Cheng Zhang and Guokang Mo contributed equally to this work. Cheng Zhang: responsible for study conception, data organization, software implementation, and preparation of the initial manuscript. Guokang Mo: contributed to study design, co‐drafted the manuscript, and undertook critical revision and editing. Yongxing Zhang: oversaw project management and supervision, assisted with conceptual planning, and supported software‐related work. Hao Xu: participated in conceptual development and provided manuscript review and editorial input. Xiangjun Hu: secured funding support for the study. Jian Zhang: involved in study design and investigation, coordinated project execution, and obtained research funding. Xiangjun Hu and Jian Zhang are co‐corresponding authors of this article. Cheng Zhang and Guokang Mo are co‐first authors for this work.

## Funding

This study was supported by the Xuhui District Institute‐Local Government Cooperation Project (23XHYD‐18) and National Natural Science Foundation of China (10.13039/501100001809, 82503091).

## Conflicts of Interest

The authors declare no conflicts of interest.

## Supporting information


**Supporting Information** Additional supporting information can be found online in the Supporting Information section. figures and tables provide additional supporting data, including reagent information (Supporting Information Table S1), primer sequences (Supporting Information Table S2), overlapping target genes (Supporting Information Table S3), molecular docking binding energies (Supporting Information Table S4), and cell viability screening results (Supporting Information Figure S1, S2). These materials supplement the experimental methods, validation, and mechanistic analysis in the main text. The graphical abstract illustrates the general workflow and key findings of the entire study.

## Data Availability

The data that support the findings of this study are available on request from the corresponding author. The data are not publicly available due to privacy or ethical restrictions.
