# Supplementary material for: Resveratrol Ameliorates Diabetic Peripheral Neuropathy via the AMPK/mTOR/Bcl‐2 Axis: Integrative Evidence From Network Pharmacology, Mendelian Randomization, and Binding‐Site Evolutionary Constraint
Source: Hum Mutat. 2026 Jun 11;2026:4524272. doi: 10.1155/humu/4524272 (PMC13255016; doi:10.1155/humu/4524272)

Graphical Abstract


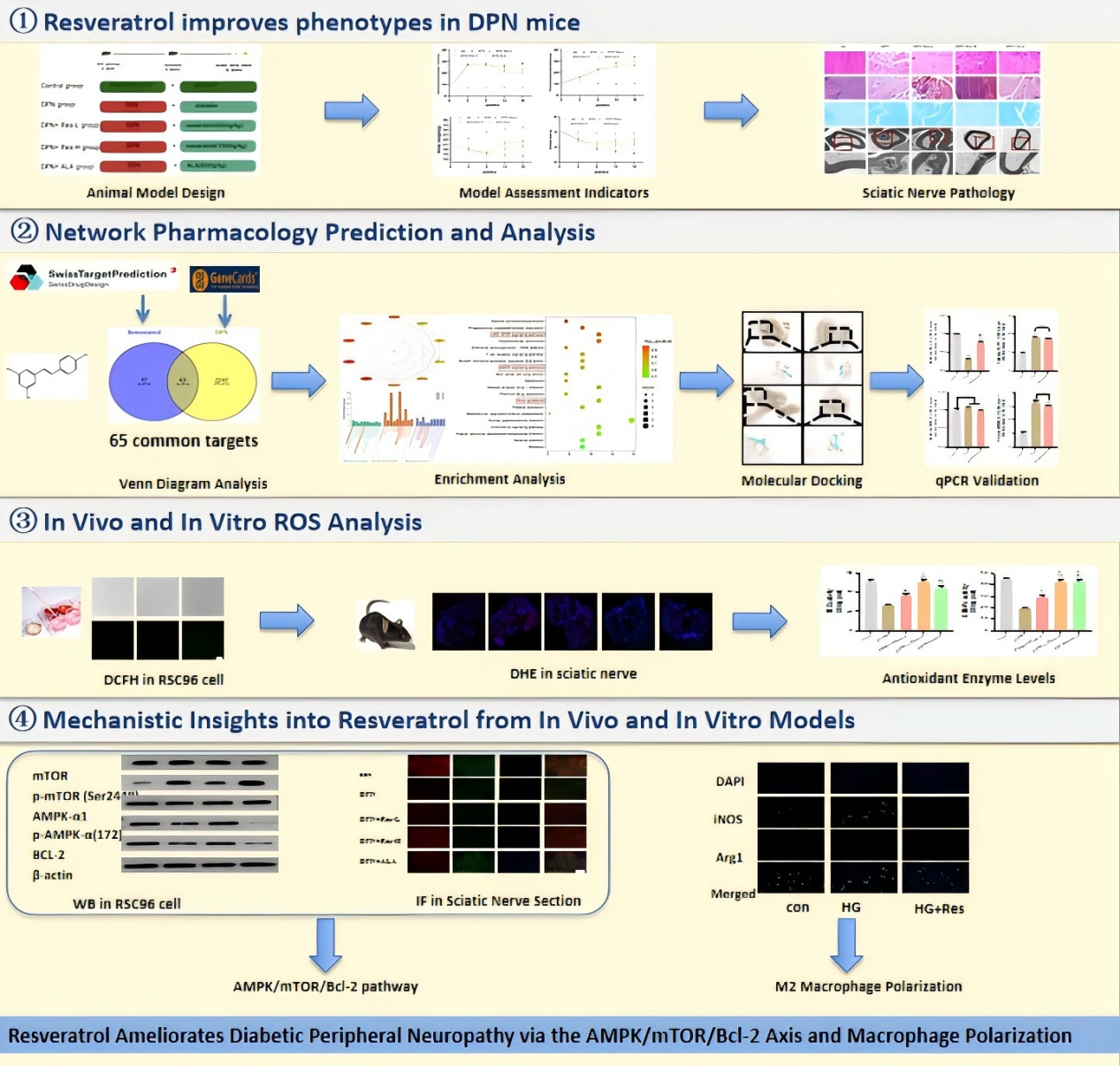


Table S1

| Reagent | Source | Catalog Number |
| --- | --- | --- |
| Resveratrol | Aladdin | R107315 |
| α-Lipoic acid | Aladdin | AL-D118666 |
| Carboxymethyl cellulose sodium (CMC-Na) | Aladdin | C104985 |
| Streptozotocin (STZ) | Sigma-Aldrich | S0130 |
| DCFH-DA | Beyotime | S1105/S0033 |
| Dihydroethidium (DHE) | Beyotime | S0064S |
| CCK-8 Kit | Beyotime | C0038 |
| TUNEL Kit | Elabscience | E-CK-A321 |
| Mouse TNF-α ELISA Kit | Nanjing Jiancheng | H052-1-2 |
| Mouse IL-10 ELISA Kit | Nanjing Jiancheng | H009-1-2 |
| SOD Activity Kit | Servicebio | GM1133 |
| GSH-Px Activity Kit | Servicebio | G4310-48T |
| Anti-Bcl-2 | Cell Signaling Technology | #4223 |
| Anti-p-mTOR (Ser2448) | Cell Signaling Technology | #5536 |
| Anti-p-AMPK (Thr172) | Cell Signaling Technology | #2535 |
| Anti-iNOS | Cell Signaling Technology | #13120 |
| Anti-Arg1 | Cell Signaling Technology | #93668 |

Table S2 PCR primer sequence

| Gene name | Prime sequence(5’ to 3’) |
| --- | --- |
| Bcl-2 | F: GGTGGACAACATCGCTCTG R: GGTCTGCAGATGCCGGTTC |
| mTOR | F: TCCGCCCTCAACAACTCC R: GAGCCATAAGCCGTTCCTC |
| MMP9 | F: CTGGACAGCCAGACACTAAAG R: CTCGCGGCAAGTCTTCAGAG |
| EGFR | F: TGCGTCTCTTGCCGGAATGT R: GGAATGCCGAGGATTGCTGA |
| β-actin | F: GGCTGTATTCCCCTCCATCG R: CCAGTTGGTAACAATGCCATGT |

Table S3 65 common elements in "Resveratrol" and "DPN"

| MAOA | CYP1B1 | PTK2B | CYP17A1 | TYMS |
| --- | --- | --- | --- | --- |
| ESR1 | CYP19A1 | ESRRA | VCP | MTOR |
| PTGS1 | TTR | IGF1R | PLAT | IGFBP3 |
| SLC6A2 | AHR | INSR | CAPN1 | NOS2 |
| PTGS2 | EGFR | KIT | DRD2 | NOS3 |
| APP | TUBB3 | SRC | PDPK1 | ADCY5 |
| PIK3CB | ABCB1 | AR | BCL2L1 | WEE1 |
| CYP1A2 | AKR1B1 | ERN1 | BCL2 | ABL1 |
| CYP2C9 | MMP9 | ESR2 | PTPN1 | FLT3 |
| CYP3A4 | MMP1 | HDAC2 | RAF1 | NOX4 |
| CA9 | MMP2 | ABCC1 | NOS1 | HTR1A |
| PIK3CA | RELA | HDAC8 | MAPT | CDK2 |
| LCK | TYR | SHBG | F3 | CDK5 |

Table S4 Best molecular docking binding energies of the top 10 genes

| Gene/protein | Best binding energy (kcal/mol) |
| --- | --- |
| MMP9 | -9.3 |
| MTOR | -7.4 |
| EGFR | -7 |
| BCL2 | -6.4 |
| SRC | -6.3 |
| ESR1 | -6.1 |
| BCL2L1 | -6.1 |
| AR | -6 |
| IGF1R | -5.6 |
| RELA | -5.4 |

Figure S1 Absorbance Values of CCK-8 Assay for RAW 264.7 Cells Cultured at Different Glucose Concentrations on Day 1, 2, and 3


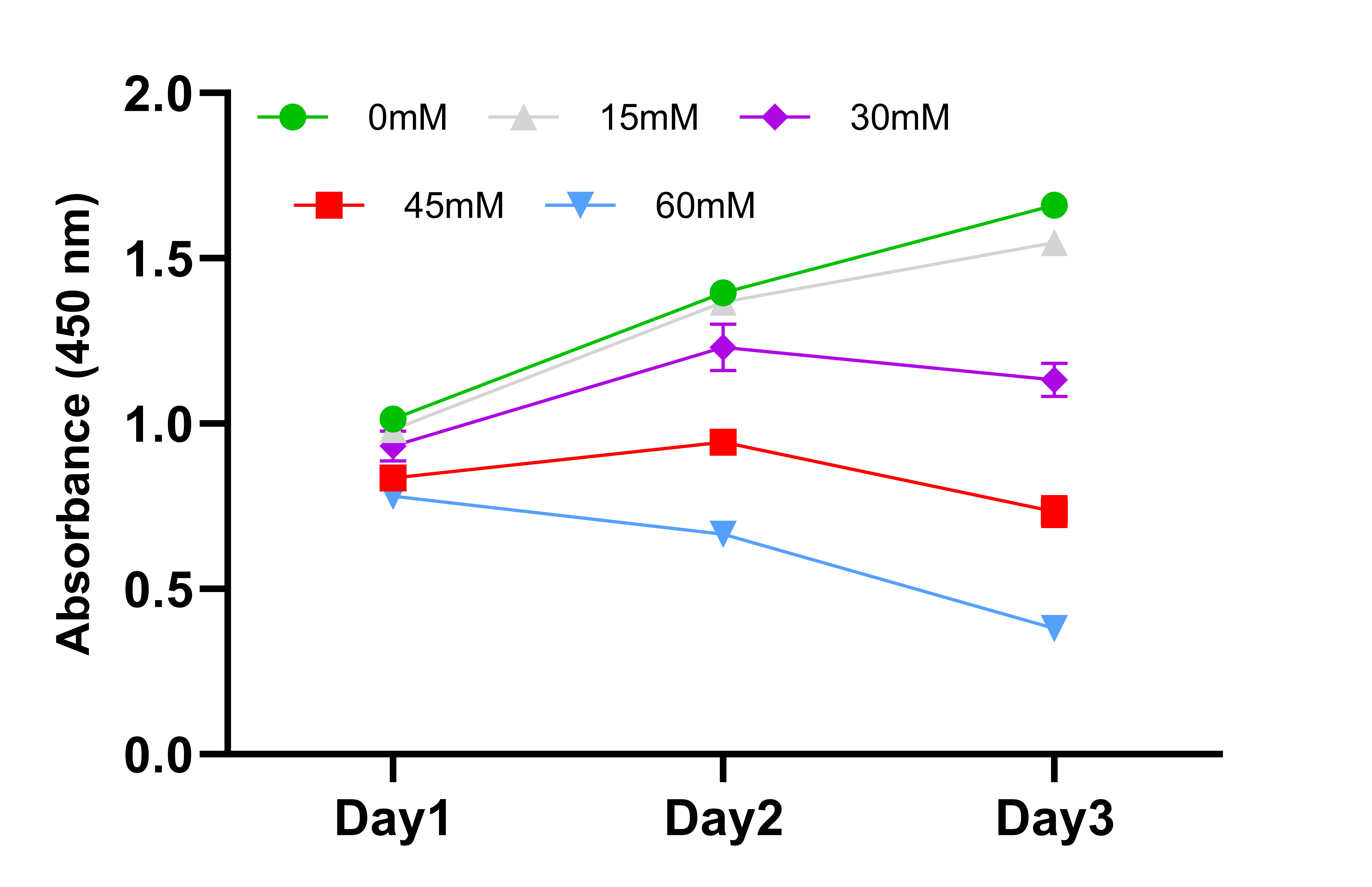


Figure S2 Absorbance Values of CCK-8 Assay for RAW 264.7 Cells Cultured at Different Resveratrol Concentrations on 24 and 48 hour.


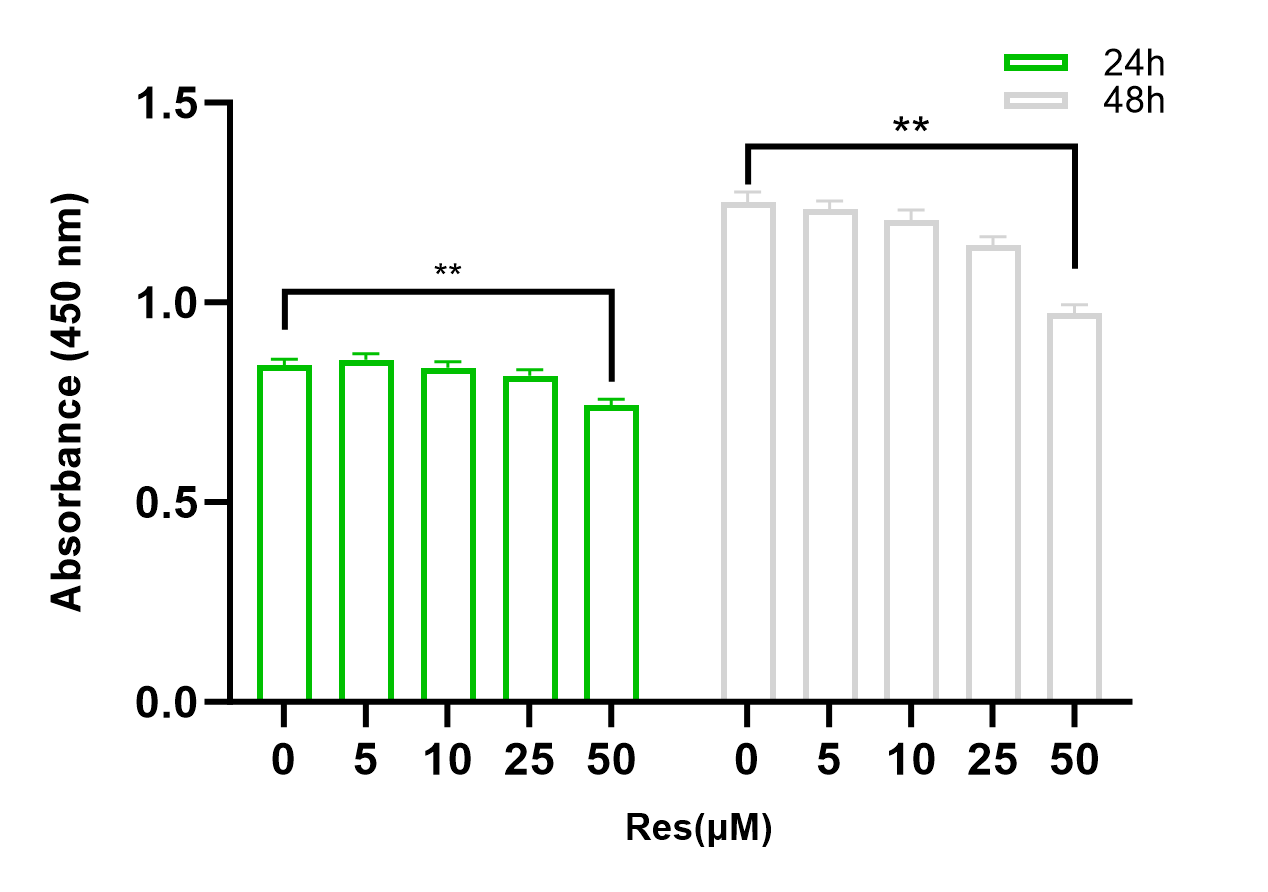

Supplement: Supplementary file 1 — Supporting Information Additional supporting information can be found online in the Supporting Information section. figures and tables provide additional supporting data, including reagent information (Supporting Information Table S1), primer sequences (Supporting Information Table S2), overlapping target genes (Supporting Information Table S3), molecular docking binding energies (Supporting Information Table S4), and cell viability screening results (Supporting Information Figure S1, S2). These materials supplement the experimental methods, validation, and mechanistic analysis in the main text. The graphical abstract illustrates the general workflow and key findings of the entire study. [file HUMU-2026-4524272-s001.docx]
